# The evolution of cheaper workers facilitated larger societies and accelerated diversification in ants

**DOI:** 10.1126/sciadv.adx8068

**Published:** 2025-12-19

**Authors:** Arthur Matte, Benoit Guénard, Shubham Gautam, Fumika Azuma, Julian Katzke, Francisco Hita Garcia, Thomas van de Kamp, Evan P. Economo

**Affiliations:** ^1^ Department of Zoology, University of Cambridge, Cambridge, UK.; ^2^ Biodiversity and Biocomplexity Unit, Okinawa Institute of Science and Technology Graduate University, Okinawa, Japan.; ^3^ School of Biological Sciences, The University of Hong Kong, Hong Kong, SAR, China.; ^4^ Center for Integrative Biodiversity Discovery, Museum für Naturkunde, Invalidenstraße, 10115 Berlin, Germany.; ^5^ Institute for Photon Science and Synchrotron Radiation (IPS), Karlsruhe Institute of Technology (KIT), 76344 Eggenstein-Leopoldshafen, Germany.; ^6^ Laboratory for Applications of Synchrotron Radiation (LAS), Karlsruhe Institute of Technology (KIT), 76131 Karlsruhe, Germany.; ^7^ Department of Entomology, University of Maryland, College Park, MD, USA.

## Abstract

Trade-offs between quantity and quality are common in the organization and evolution of biological, technological, and economic systems. In social insects, shifts from solitary organisms to complex societies bring this dilemma to the colony scale: producing fewer robust units or many cheaper ones. We investigate how cuticle investment, a major nutritional cost, shaped the evolution of ant societies and diversification. Using a computer vision approach on three-dimensional x-ray microtomography scans of 880 specimens from 507 species, we show that larger colonies were facilitated by reducing exoskeleton investment rather than miniaturizing workers. Reduced cuticle investment was associated with accelerated diversification rates in ants, whereas other candidates—colony size and worker size—did not correlate with diversification. Diet and climate had measurable but secondary effects on cuticle investment. Our results support a hypothesis whereby evolving cheaper but more numerous units through reduced investment in structural tissues was a strategic trend in the evolution and diversification of complex insect societies.

## Teaser

Ants rose by favoring the power of many over the might of few.

## INTRODUCTION

Trade-offs between quantity and quality are ubiquitous across various domains. Organisms must typically balance between producing numerous inexpensive offspring or investing in higher quality but fewer progeny, a strategic problem shaping the evolutionary trajectories of countless species (*1*). The evolutionary transition from solitary organisms to complex superorganisms in social insects brings this trade-off to a new level: the structure of the society itself. Should a colony produce and maintain a workforce of fewer, high-cost individuals or a larger number of cheaper ones?

Larger societies enable more complex social and behavioral organization (*2*–*4*), facilitating collective defense against pathogens and predators (*5*, *6*). Larger colonies are also more resilient to environmental fluctuations (*7*) and infections (*8*, *9*). Furthermore, larger colonies can more effectively defend their territory and resources against competitors (*10*). In principle, a colony can build more individuals by making them smaller, packing resources into smaller units, or investing less in costly tissues, such as cuticle (*11*, *12*).

Cuticle represents a substantial investment in nitrogen and other scarce elements such as zinc and manganese (*13*, *14*). Despite its high cost, the cuticle is essential for insect survival, serving as the primary protective barrier against predators, pathogens, mechanical shocks, harmful radiation, and desiccation while also supporting muscle attachment and thermoregulation (*14*–*21*).

As globally distributed and ecologically dominant organisms (*22*), ants demonstrate an extraordinary range of ecological niches and social complexities (*4*, *23*, *24*). Cuticle thickness exhibits considerable variation among ant species, ranging from 1.3 to 109.8 μm in previous comparative analyses (*12*, *21*). This variability suggests that some species favor thicker cuticles for greater protection, while others prioritize thinner cuticles to cope with nutrient scarcity or allocate resources elsewhere. Previous analysis focusing on other defensive traits including sting, dorsal spines, eye size, and colony size suggested them to be associated with diversification rates (*25*). Despite the apparent physiological and ecological significance of cuticle investment and its important variations across species (*12*, *21*), macroecological and evolutionary studies across clades are still lacking. Specifically, large-scale patterns of variation have not been quantified, and the specific factors driving its variation remain to be understood. A macroscopic analysis in cuticle investment could reveal how social insects optimize resource allocation in response to their environmental and social contexts.

Recent advances in high-throughput microtomography techniques have enabled considerable scanning efforts of small arthropods (*26*), providing an unprecedented opportunity to study large-scale anatomical traits, such as cuticle evolution (*27*), in a comprehensive manner. Here, we introduce a method for unsupervised segmentation of 3D-scanned specimens and analyze data from more than 880 ant scans. Through phylogenetic comparative methods, we aim to untangle the evolution of cuticle investment in ants and its relationship with ecological, social, and diversification factors.

## RESULTS AND DISCUSSION

**Fig. 1.**
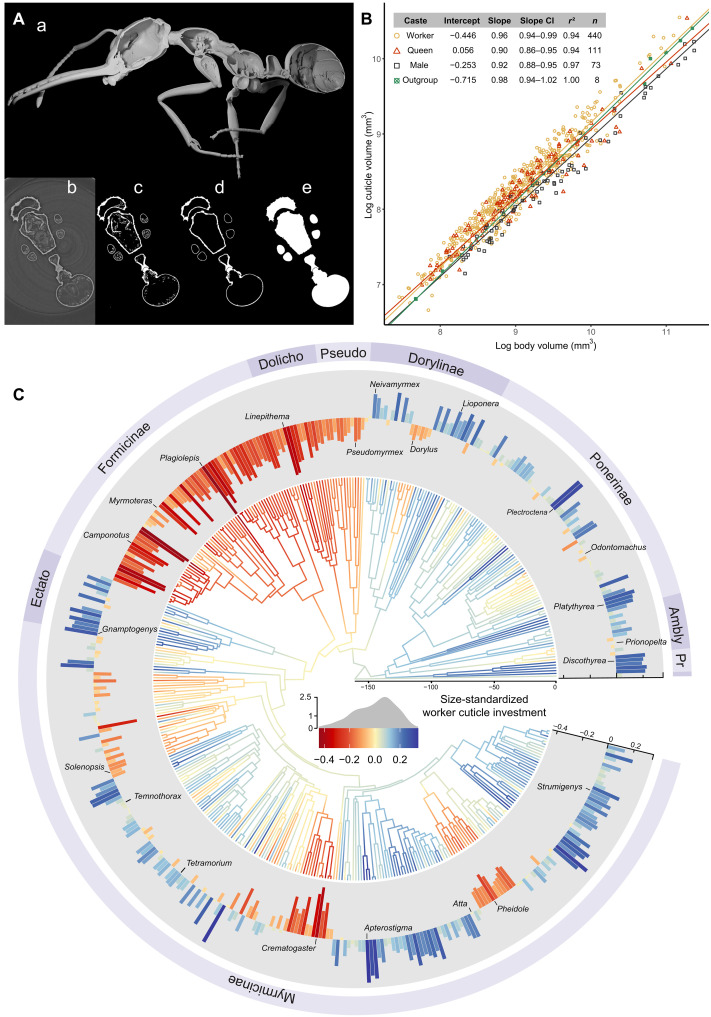
Evolution of cuticle investment in ants. (**A**) Main steps of our program for unsupervised segmentation of the cuticle and body volume. (a) Sagittal cut of a *Myrmoteras* ant worker with cuticle segmented by our unsupervised program. Starting from the original slice (b), image colors are adjusted and the vial is detected and cropped. We then apply a pixel intensity threshold (c) and filter contours inside another to extract cuticle volume (d). We lastly fill cuticle contours to extract body volume (e). These steps were applied to all image slices composing the scans to obtain the global cuticle and body volume. (**B**) Allometric relationship between cuticle volume and body volume in ant workers, queens, and males compared to the outgroup. Each data point represents the average cuticle volume and body volume per species per caste. The table summarizes the regression parameters obtained from SMA regression. CI, confidence interval. (**C**) Ancestral state reconstruction of the size-standardized worker cuticle investment for 434 species (174 genera). Colored branches indicate predicted values of traits, and the density plot reflects the density of tip values. Time is expressed in Ma, and major subfamilies are labeled as follows: Ambly, Amblyoponinae; Dolicho, Dolichoderinae; Ectato, Ectatominae; Pr, Proceratiinae; Pseudo, Pseudomyrmecinae. Warmer (redder) colors represent decreasing cuticle investment, whereas cooler colors (darker blues) represent higher cuticle investments. Key genera are provided as landmarks, and a similar tree with full details of species as well as ancestral state reconstructions for queens and males is provided in fig. S2.

We developed a computer vision–based method to automate the segmentation of bodies and cuticles from x-ray microtomography data (Fig. 1A). This approach enabled the extraction of cuticle and body volumes from three-dimensional (3D) images of 880 specimens. Our sample included 655 ant workers (440 species:178 genera), 136 ant queens (111:63), 80 ant males (73:52), and 9 specimens from six other Hymenoptera families, Bethylidae, “Chalcidoidea/Chrysidoidea,” Polistinae, Scoliinae, Sceliphrinae, and Pompilidae, used as outgroups.

### Allometric body size scaling and caste-specific differences

Cuticle volumes are expected to primarily scale with body volume, following allometric patterns as observed in other groups. Skeletal structures typically exhibit near-isometric scaling in insects (*28*) and hyperallometry in mammals (*29*). Therefore, to analyze variation in cuticle investment, we first need to establish a baseline size scaling.

We examined the interspecific allometric scaling between cuticle volume and body volume in ants and compared them to an outgroup of nine flying hymenopterans using standardized major axis (SMA) regression on log-transformed data (Fig. 1B). In ants, the cuticle volume exhibited hypoallometric scaling, increasing at a slightly slower rate than body volume (test against isometry: *P* < 0.01 for all ant castes). Among ant castes, workers exhibited the scaling rate closest to isometry (slope_workers_ = 0.96 ± 0.03), whereas queens and males had significantly more hypoallometric scaling (slope_queens_ = 0.90 ± 0.04, slope_males_= 0.92 ± 0.04, both different from workers, *P* < 0.05). No significant differences in slope were detected between queens and males (*P* = 0.66). We additionally tested for differences between worker subcastes, focusing on a set of species with data for both minor and major workers, but found no significant difference in slope (*P* = 0.13, *n* = 41).

In contrast, flying hymenopterans exhibited isometric scaling (test against isometry: *P* = 0.5), aligning with previous findings (*28*) but also potentially due to limited sample size. Lease and Wolf (*28*) previously noted lower allometry in flying insects compared to nonflying ones. Similarly, our observation for lower allometry in reproductive castes of ants (typically winged) compared to their wingless worker castes suggests a flight-dependent polyphenic divergence in cuticle investment between ant castes.

To estimate cuticle investment while accounting for body size, we used the residuals from the SMA regression of log_10_(cuticle volume) against log_10_(body volume) using species-averaged values for each caste. We then compared size-standardized cuticle investment across castes within species (fig. S1). Worker cuticle investment showed correlations with that of queens (*r*^2^ = 0.63, slope = 1.06, *P* < 0.001, *n* = 74) and with that of males (*r*^2^ = 0.64, slope = 0.87, *P* < 0.001, *n* = 45). However, cuticle investments of queens and males were not significantly correlated in our limited sample (*r*^2^ = 0.15, *P* = 0.10, *n* = 20). The relatively low correlation coefficients between workers, queens, and males likely reflect the distinct selective pressures shaping cuticle investment in each caste.

Cuticle variations were more explained by body volume in males (97%) than in queens and workers (both 94%; Fig. 1B). This difference between sexes may arise from the diverse ecological and life-history traits shaping female exoskeletons (see below), whereas males of nearly all species share relatively similar life histories, typically protected within the nest until a brief mating flight before death (*30*), although not systematically (*31*, *32*).

### Macroevolution of cuticle investment

#### Ancestral state reconstruction and patterns of evolution

Cuticle investment in ant workers has undergone remarkable shifts throughout ant evolution. In our dataset, *Apterostigma megacephala* and *Plagiolepis afrc-gh01* exhibited the highest and lowest size-standardized cuticle investment, respectively. In these species, cuticle volume accounted for 35 and 6.6% of total body volume, respectively (Fig. 1C). Ancestral state estimations revealed marked reductions in size-standardized worker cuticle investment across multiple lineages, including Formicinae, Dolichoderinae, Pseudomyrmecinae, certain Mymicinae, and some Dorylinae (Fig. 1C). Phylogenetic modeling supports the notion that the evolution of this trait best fits a Brownian motion model, with a λ phylogenetic signal of 0.91 (table S1). This indicates that cuticle investment evolved in a continuous and trend-free manner over time, with trait variations reasonably phylogenetically conserved (*33*).

Bayesian reversible-jump Markov chain Monte Carlo (MCMC) identified several substantial shifts in cuticle investment, notably at the roots of Formicinae, Pseudomyrmecinae + Dolichoderinae, Stenammini, *Crematogaster*, and *Pheidole*, all reflecting reductions in cuticle investment relative to the ancestral state. The most pronounced shift occurred in Formicinae (fig. S3). To assess the robustness of this finding, we repeated the analysis using a recently published genus-level phylogeny (*34*). This alternative approach detected an initial shift toward lower cuticle investment at the node uniting Formicinae, Dolichoderinae, and Pseudomyrmecinae, followed by a reversion toward ancestral levels at the base of Myrmicinae + Ectatomminae (fig. S4). Although representing each genus by a single species may have affected shift detection in this second analysis, our results consistently support major reductions in cuticle investment within Formicinae, Dolichoderinae, and Pseudomyrmecinae. Reductions in other subfamilies appear to be more localized in specific tribes or genera.

We hypothesize that variations in worker cuticle investment during ant diversification are shaped by a combination of social, ecological, and morphological factors. Specifically, we predict a negative correlation between colony size and cuticle investment, reflecting a potential trade-off between individual quality and quantity (*11*, *12*). Given the influence of resource availability on cuticle investment, we further expect cuticle thickness to vary across dietary classes, with differences among predatory, omnivorous, herbivorous, and fungivorous species (*25*). Recognizing the role of the cuticle in preventing desiccation, we expect cuticle investment to correlate with climatic variables, predicting thicker cuticles in regions with higher temperatures and lower precipitation (*20*, *21*). Similarly, foraging microhabitats, which influence exposure to environmental stressors (*35*), may shape cuticle investment needs. To test this, we compared cuticle thickness across arboreal, epigeic, and hypogeic species. Last, we hypothesize that dorsal spines, a morphological defensive trait (*36*, *37*), may either complement or substitute higher cuticle investment, reflecting alternative evolutionary strategies against predation.

**Fig. 2.**
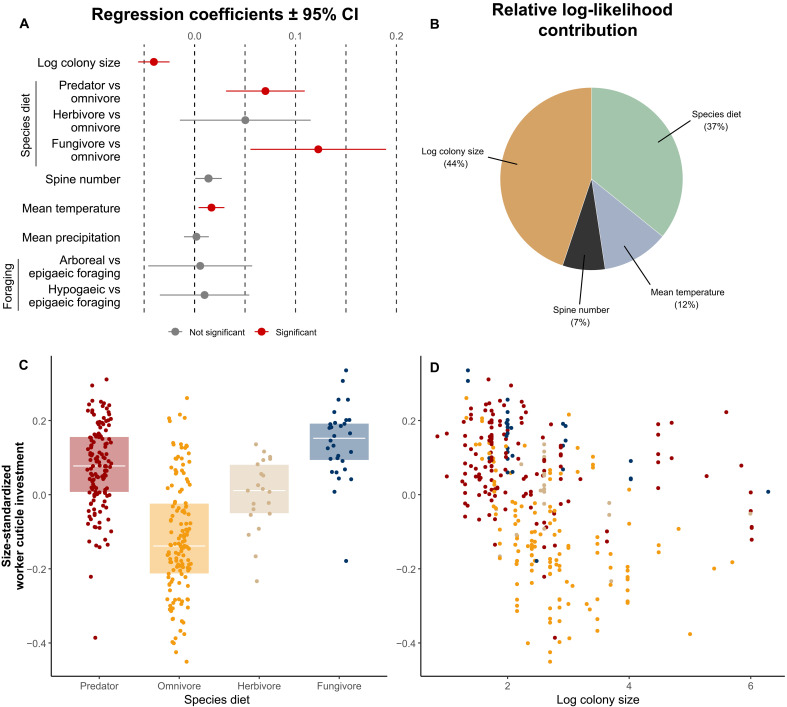
Comparative analysis of variables correlated with size-standardized cuticle investment in ant workers. (**A**) Regression coefficients and 95% confidence interval variable tested in a PGLS model including 325 species. Red dots indicate significance. (**B**) Relative log-likelihood contribution (in percent) of significant variables within our PGLS model. (**C**) Worker size-standardized cuticle investment according to species diets and (**D**) log colony size. Dots were colored according to species diets. Size-standardized cuticle investment was calculated as the residuals from the SMA regression of log_10_(cuticle volume) against log_10_(body volume). The effect of body size on absolute cuticle volume, included as a covariate to account for the allometry, has not been represented from the figure and log-likelihood contribution calculation.

We used a phylogenetic generalized least-square (PGLS) analysis to account for the phylogenetic dependence of our taxa in a dataset comprising 325 species spanning 133 genera and 14 subfamilies (fig. S5). PGLS results are summarized in Fig. 2, with detailed values available in table S2 (and table S3 for sensitivity analysis).

Our analysis revealed a strong negative correlation between colony size and size-standardized cuticle investment (*P* < 0.001). Among the variables tested, colony size contributed the most to the explanatory power of our model, accounting for 44% of the total log-likelihood attributed to the model with all variables tested together (Fig. 2, A and B). Beyond this core relationship, cuticle investment was higher in predator and fungivore species compared to omnivores (both *P* < 0.001) and in species inhabiting warmer regions (*P* < 0.005). The presence of dorsal defensive spines was marginally positively correlated with higher cuticle investment (*P* < 0.052) (Fig. 2C).

#### Cheaper workers and colony size

Larger colonies come with several social consequences that likely reduce the need for robust exoskeletons. These outcomes include diluted individual predation risk; enhanced collective defense against pathogens, parasites, and predators (*5*, *6*, *8*, *9*); and increased social complexity (*4*). In addition, larger colonies benefit from more collective foraging strategies (*2*), improved task organization (*3*), and greater abilities to defend their territory and resources against competitors (*10*).

On the other hand, while reduced cuticle investment compromises the protection of workers, it concurrently liberates nitrogen resources previously dedicated to cuticle formation. This shift may support the development of other critical organs, such as muscles and the brain, but it can facilitate the production of additional colony members, as highlighted by Nalepa (*38*) and Peeters *et al.* (*12*). This dual phenomenon suggests a positive feedback loop: Reduced exoskeletal investment enables larger colonies, and in turn, larger colonies reduce the need for individual protection. In this context, the lack of overt defenses in workers would be compensated by their high production rate, making each worker individually more expendable, as proposed by Peeters *et al.* (*11*, *12*).

**Fig. 3.**
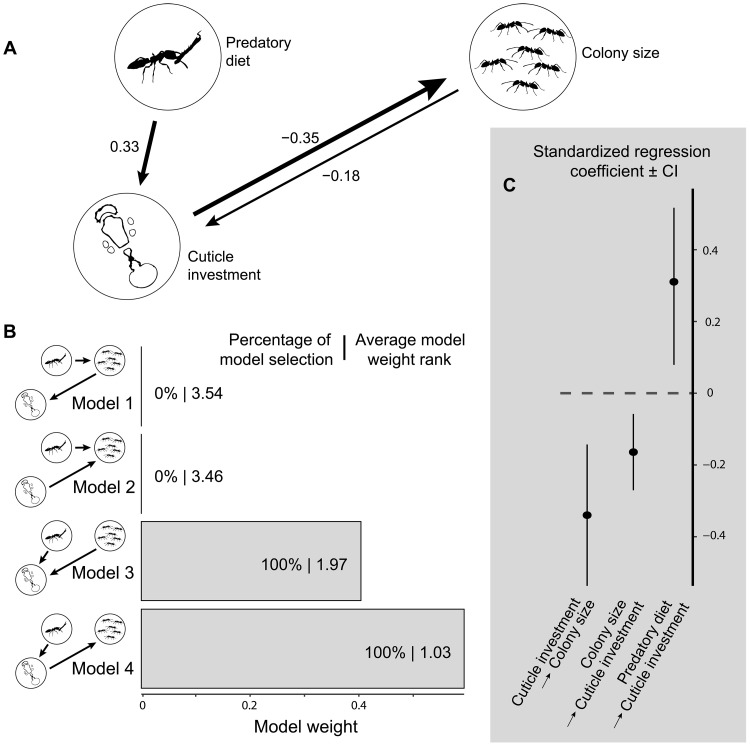
Causal inference of relationships across species diet, size-standardized worker cuticle investment, and colony size. Multitree phylogenetic path analysis performed on a dataset of 172 species (103 genera) with species-level data and run over 200 phylogenetic trees. (**A**) Causal relationships between variables from averaging the best models (model 3 and 4). Arrow values indicate phylogenetic path coefficients. (**B**) Model weight estimation and comparison over multiple phylogenies with details of the percentage of model selection (best models within a range of 2 CICc) and average model weight rank. *P* values (whose significance indicates model rejection) were below 0.05 for models 1 and 2 and more than 0.6 for models 3 and 4. (**C**) Standardized regression coefficients ± confidence interval estimated over multiple phylogenetic trees from averaging the best models (models 3 and 4).

To disentangle the causal relationships between cuticle investment and colony size, we constructed four plausible evolutionary models reflecting different hypotheses for causal relationships between cuticle investment, colony size, and diet (Fig. 3). The likelihoods of the models were compared over 200 phylogenies using phylogenetic path analysis. We found that models where cuticle investment drives colony size consistently outperformed models where colony size drives cuticle investment. In addition, cuticle investment had an overall stronger effect on colony size than the reverse relationship. However, the difference in support between cuticle- and colony size–driven models was consistently small [always less than 2 CICc (C-statistic information criterion corrected for small sample size); Fig. 3B], suggesting that worker cuticle investment and colony size are deeply interwoven.

The lack of physical defenses in some ant species may be offset by alternative protective strategies, which are more viable in larger colonies. For example, poison sprays or volatile chemicals (as seen in Dolichoderinae and Formicinae) do not require direct physical contact, in contrast to ancestral stinging mechanisms relying on close engagement. This draws an interesting analogy with historical changes in human warfare, where heavily armored knights gave way to archers and crossbowmen who needed far less protection (*39*). Myrmicinae, which exhibits reduced cuticle investment, also developed a “knight” caste, where major workers take on protective roles (e.g., *Pheidole* and *Solenopsis*) (*40*), while *Crematogaster*, lacking functional stingers, rely on contact or volatile poison strategies. Investigating the coevolution between cuticle investment and alternative defense mechanisms in ants would be a promising avenue for clarifying these relationships.

#### Worker body volume and colony size

Peeters and Ito (*11*) proposed that smaller workers might facilitate larger colonies by packing resources into smaller units. We tested this hypothesis using our dataset pruned of majors and media, calculating the mean body volume of the smallest workers per species. Contrary to expectations, larger colonies were associated with larger workers, regardless of whether we considered data at the genus or species level and whether we accounted for phylogeny (all but one *P* < 0.05, table S4). We included the mean annual temperature in the model to account for any potential confounding effect of body size and climate [e.g., Bergmann’s rule (*7*, *41*, *42*)], but the mean temperature showed no correlation with body volume in ants (all *P* > 0.3, table S4). These findings, along with our previous results, support the notion that colony size increases are primarily correlated with reducing cuticle investment rather than worker miniaturization.

#### Diet

Building on ants’ ancestral predatory diet (*43*), the evolution of novel nutritional strategies has played a critical role in shaping cuticle investment during ant diversification. Predator and fungivorous species typically exhibit thicker cuticles compared to omnivorous species, which tend to have thinner ones (Fig. 2, A to C). This pattern is likely driven by the higher nitrogen content of prey- and fungus-based diets relative to plant-based diets (*44*, *45*). Noteworthily, some species with nutrient-poor diets could also achieve thicker cuticles through symbiotic nitrogen-providing organisms [e.g., *Cephalotes* (*46*)].

Certain atypical strategies, such as those used by the leaf-cutting fungus-growing ants (*47*, *48*) and the highly predatory Dorylinae army ants (*49*), appear to support the evolution of massive colonies numbering millions of individuals while still maintaining reasonably armored workers (Fig. 1C). However, even within these lineages, the general trend of reduced cuticle investment with increasing colony size persists (e.g., *Apterostigma* versus *Atta*; *Neivamyrmex* versus *Dorylus*; Fig. 1C). This highlights the ubiquity of the trade-off between cuticle investment and colony size, although its magnitude is modulated by specific nutritional strategies.

#### Foraging microhabitats

The greater cuticle thickness in insects has been proposed to be adaptive against desiccation (*20*, *21*). Arboreal ants are more exposed to heat and desiccation stress (*35*, *50*) and are often found to have darker cuticles compared to terrestrial ants (*51*). However, our study detected no significant effect of arboreal foraging on cuticle investment, nor did we observe any effect of hypogean foraging, where ants are generally less exposed to desiccation stresses (Fig. 2C). While variation in cuticle investment as a result of foraging microhabitats may exist within genera, intergeneric variations were not substantial enough to be detected in this broad-scale analysis. Furthermore, cuticle thickness is not necessarily the main barrier against desiccation; this resistance may primarily lie in epicuticle hydrocarbons (*52*, *53*) and even in behavioral adaptations, such as nocturnality or seeking cooler, wetter environments [common in small organisms like ants (*54*)].

#### Climate

We found that species living in regions with higher mean annual temperatures tend to exhibit higher cuticle investment. Although the regression coefficient was relatively small, this effect was statistically significant and accounted for 12% of the model likelihood (Fig. 2, A and B). This association may be attributed to increased pathogen stress in warmer regions (*55*, *56*). Thicker cuticles are thought to play a crucial role in protecting against pathogen infections, particularly fungi, which must breach the cuticle to infect the individual (*18*, *57*).

Overall, our analysis supports the notion that the evolution of larger societies was facilitated by the development of workers with less expensive exoskeletons, although worker size did not decrease. Diet and climate had measurable but secondary effects. Intrageneric or even intraspecific studies would be necessary to gain a deeper understanding of how climates and microhabitats influence cuticle thickness in ants.

#### Sensitivity analysis

To assess the robustness of our findings across alternative phylogenetic frameworks, we repeated the PGLS and phylogenetic path analyses on the basis of a recently published tree (*34*). Because this tree contains one species per genus, we ran our PGLS analysis with a matching dataset of 128 species instead of the original 325. Despite the smaller sample size, all key correlations remained consistent with our primary analysis. Specifically, the strong negative correlation between colony size and cuticle investment persisted (*P* < 0.001), along with significant effects of predator and fungivore diets (table S3). However, the weaker correlations of cuticle investment with mean annual temperature (*P* = 0.10) and with spine number (*P* = 0.21) were no longer supported, possibly due to the reduced sample size. No new correlations emerged.

In addition, we performed the phylopath analysis using this newer phylogeny with a matching dataset of 97 species. The best-supported causal models and coefficients remained nearly identical to those shown in Fig. 3, maintaining the inference that cuticle investment exerts a stronger effect on colony size than vice versa. These results confirm the stability of our conclusions across alternative phylogenetic hypotheses and methods.

### Cuticle investment and diversification rates

**Fig. 4. Reduced cuticle investment accelerated diversification rates.**
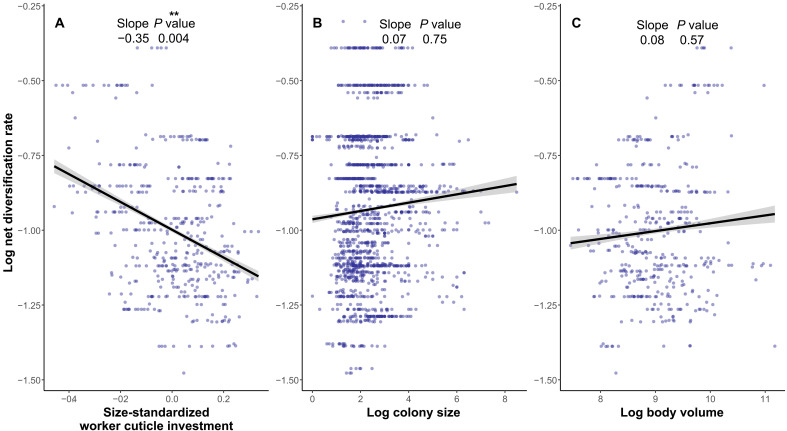
Ant net diversification rates versus (**A**) size-standardized cuticle investment, (**B**) colony size, and (**C**) body volume. Consensus diversification rates were calculated as the mean rates from six different methods. Details of the variations across these methods are provided in fig. S6 and table S5.

Investigating trait-dependent diversification revealed a compelling link between reduced cuticle investment and higher diversification rates (Fig. 3A). This relationship was consistently observed across various dating and tree reconstruction methods, regardless of whether we accounted for undiscovered ant diversity through clade grafting or incomplete sampling fractions (Fig. 4, table S5, and fig. S6). Specifically, cuticle investment exhibited a significant negative correlation with diversification rate in four of the six methods used to estimate diversification rates (ES-sim; mean rho: −0.34 ± 0.07; all *P* < 0.02, *n* = 434). The remaining two methods approached significance (both *P* < 0.07).

Given the established correlation between cuticle investment and colony size (see above) and the proposed link between colony size and diversification rates (*25*, *58*), we further explored this relationship. However, our analysis found no association between colony size and diversification rates (all *P* > 0.5, *n* = 1616; Fig. 4). We also examined the hypothesis that ant success might derive from reduced worker production costs via body size reduction, independent of cuticle investment (*11*). Our analysis, however, revealed no significant association between body volume and diversification rates (all *P* > 0.35, *n* = 424; Fig. 4). Details of values and correlations across methods are provided in table S5 and fig. S6.

Our results support the notion that the social and ecological innovations associated with reduced cuticle investment have been fruitful strategies for ants, leading to increased diversification rates. Although colony size played a role in this narrative, it is not the sole factor as it was not linked to diversification rates itself. Species able to cope with weaker exoskeletons can also better perform in nutrient-poor environments by needing less nitrogen (*44*). Key factors such as the degree of social organization among workers, regardless of colony demographics, that enhance social buffering, as well as the claustral colony foundation enabled by cheaper workers (*59*), would be promising avenues to better understand the evolutionary success of ants with reduced cuticle investment.

### Strategic cost reduction and the rise of ant societies

Our study provides a macroscopic view of the evolution of cuticle investment in ants, a key parameter of the ant phenotype, and highlights the potential of high-throughput 3D image generation and analysis for large-scale trait analysis. Our findings support the hypothesis that the evolution of larger societies was facilitated by the development of workers with less costly exoskeletons, although not necessarily smaller individuals. Diet and climate had measurable but secondary effects. Our findings support the notion that reduced cuticle investment is associated with accelerated diversification rates in ants, whereas neither colony size nor worker size showed such an effect in isolation. This suggests that the evolutionary success of ants with reduced cuticle investment stems from a combination of social and ecological innovations, including cost-effective worker production and adaptive social organization. The ability to thrive with less costly exoskeletons allows ants to exploit nutrient-poor resources while benefiting from reduced worker production costs. Collectively, our results provide a deeper understanding of how cuticle investment, dietary factors, and environmental conditions interact to shape the evolutionary trajectories of ants. These findings highlight the complex interplay between social organization, morphology, ecology, and evolutionary success.

Many questions remain to be explored using our dataset and approach. Future research could examine how cuticle investment reflects the metabolic division of labor and symbiotic partnerships. Investigating its variation across body parts may shed light on the pressures shaping exoskeleton development. Exploring its interplay with other costly traits, such as brain size and reproductive capacity, could reveal additional aspects of the “cheaper worker syndrome.” More broadly, our findings challenge conventional views on social evolution, highlighting how strategic reductions in individual investment can unlock large-scale adaptive success. By revealing the evolutionary blueprint of cost-efficient worker production, this work paves the way for a new understanding of how biological systems optimize function, resilience, and dominance within ants and across the tree of life.

## MATERIALS AND METHODS

### Data sampling

We developed a program for unsupervised segmentation of 3D-scanned specimens and estimation of the cuticle and body volumes. Using scans from the Antscan database (www.antscan.info) (*26*), our program allowed us to obtain data for 880 specimens encompassing 655 ant workers (439 species:178 genera), 136 ant queens (111:63), and 80 ant males (73:52). We included nine specimens from six other Hymenoptera families (Bethylidae, “Chalcidoidea/Chrysidoidea,” Polistinae, Scoliinae, Sceliphrinae, and Pompilidae) used as an outgroup. We analyzed the evolution of cuticle investment and its relationship with a panel of morphological, ecological, and social traits. In our analysis as well as in our data collection, all species names were updated according to the latest taxonomic revisions (AntCat.org, released September 2023). Ant workers of the Antscan database are typically foragers, minimizing the risk to analyze specimens whose cuticle is not fully developed yet (*60*).

### Sample size variation across analyses

Sample sizes varied across analyses because of differences in data availability for specific traits and analytical requirements. The allometric scaling analysis included all species with both cuticle and body volume measurements (*n* = 632). Ancestral state reconstructions were restricted to worker specimens with available phylogenetic placement (*n* = 434). The PGLS analysis was conducted on species with complete data for all tested predictors—including mean annual precipitation, mean annual temperature, colony size, spine number, diet, and foraging microhabitat—resulting in a more limited dataset (*n* = 325). Phylogenetic path analysis required species-level colony size estimates, further reducing the sample size (*n* = 172). Diversification analyses used species with phylogenetic placement and trait data relevant to each tested variable: worker cuticle investment (*n* = 434), nest size (as a proxy for colony size; *n* = 1616), and worker body volume (excluding majors and media; *n* = 424).

### Synchrotron x-ray microtomography

All specimens originate from the Antscan repository (*26*). Synchrotron x-ray microtomography was performed at KIT Light Source. For each scan, 3000 radiographic projections with a 2016- by 2016-pixel resolution were taken and tomographically reconstructed, resulting in a 3D image (tomogram), which formed the basis for data analysis here. Depending on their size, ants were scanned with magnifications of 10×, 5×, or 2×, resulting in effective pixel sizes of 1.22, 2.44, or 6.11 μm, respectively. In cases where the specimens exceeded the field of view, additional height scan steps were conducted to ensure complete coverage. The method is fully detailed by Katzke *et al.* (*26*).

### Cuticle and body volume measurements

The manual segmentation process commonly used to extract quantitative trait data from x-ray tomograms is both labor-intensive and time-consuming and not feasible for many hundreds of scans. In response to this challenge, we developed an automated segmentation program that aligns with the broader goal of harnessing microtomography for large-scale comparative morphology studies (Fig. 1A). Our approach performs efficient serial 2D computations to analyze 3D scans, requiring minimal random-access memory and computational power. As a result, the program runs smoothly even on standard laptops equipped with Python. While artificial intelligence–driven segmentation might dominate future workflows, our program offers viable alternative and complementary tools for studies requiring precision without training data.

Using computer vision libraries (*61*) in Python version 3.10.5, we implemented logical rules tailored to the morphological characteristics of insect cuticle, enabling specific segmentation of this tissue. This approach relies solely on contour detection and logical filters without the use of artificial intelligence. Detailed procedures are provided in the scripts in the Supplementary Materials and summarized below.

We analyzed each 2D image slice of a tomogram separately to retrieve the number of pixels corresponding to the cuticle and the full body on each. Once the thousands of images composing the 3D volumes were processed, we summed the results and multiplied it by cubed pixel size to obtain the total cuticle volume and total body volume of specimens. To specifically segment the cuticle, our program relies on the fact that the cuticle is always the outermost tissue of the specimens. The program then fills up the cuticle contours to estimate the body volume.

Our program works as a decision tree in which we implement different rules to handle and filter the various contours detected on 2D images. Initially, the contrast of the 2D slices was enhanced to facilitate segmentation: sharpening edges and reducing blurriness by keeping the edges using a bilateral filter. Given that specimens were scanned in ethanol-filled vials, we detected the border of the vial and cropped it to prevent interference in subsequent steps. Then, a broad pixel intensity threshold was applied to retain pixels within a specified range (Fig. 1Ab). Given that the cuticle is always the outermost tissue of the specimens, we extracted and filtered the contours of the segmented images according to the contour hierarchy to retain only the external contours and their corresponding internal contours (Fig. 1Ac). In other words, contours inside another contour but corresponding not to its internal border were removed. Extremely small contours with perimeters fewer than 40 pixels were removed as well. In some species, the cuticle of some body parts (typically the gaster) is so thin that segments appear not fused, leading to open exoskeleton contours. In such cases, a convex hull was drawn around these contours—detected as orphan childless—to artificially close them and remove their internal contours. For each segmented slice, the cuticle volume was calculated by summing the cuticle pixels, while the body volume was obtained by summing the pixels after filling each close contour with white (Fig. 1Ad). When the cuticle edges were not closed, we applied further steps to link contours together into a closed exoskeleton, or in the case of failure of these steps, we approximated them as close convex hulls.

Because of the nature of our approach, the quality of our automated segmentation ranged from exceptional results, potentially surpassing the accuracy of manual segmentations, to failures arising from different factors (e.g., insufficient contrast between tissues and inadvertent segmentation of the vial). To ensure the integrity of our dataset, we visually inspected all the segmentation images generated by our program and removed erroneous specimens.

To further mitigate punctual imperfections in the image series segmentation, we anticipated linearity in the extracted results from consecutive slices to apply a rolling median filter to the lists of extracted body and cuticle volumes to remove punctual errors. In other words, errors occurring on a limited range of segmented images, framed by good-quality segmentation, will not affect our results as they are adjusted to fit the continuity in the value list.

Pixel threshold values were initially similar for all segmentations, but when necessary, we adjusted the segmentation threshold values to optimize results according to the specifics of each scan. In addition, we segmented parts of the bodies of 48 specimens under different programs tailored to deal with specific situations such as disjointed gaster segments. The final extracted values for each scan along with the segmented images are available in the Supplementary Materials.

Many ants in the Antscan database were imaged in height steps because of their size exceeding the vertical field of view. As we worked with an early version of Antscan that did not yet include stitching, individuals split across multiple folders were segmented independently before being reassembled. To merge extracted data from multiple scans while accounting for overlap, we applied a contig-like reconstruction approach. Given that the extracted scan data formed long lists of values matching image succession, we aligned consecutive lists and identified overlapping regions on the basis of minimal variation in extracted values for both cuticle and body volumes.

To validate the accuracy of our automated segmentation program, we manually segmented 14 individuals and estimated their cuticle and body volumes using Amira 2019.2. Species were selected to represent diverse ant subfamilies, body sizes, and morphologies, allowing us to evaluate model accuracy across broad morphological variation. We compared the volume of the cuticle and the ratio of cuticle volume to body volume obtained from both methods, yielding strong correlations (*r*^2^ = 0.996 and *r*^2^ = 0.969) and linear relationships, strongly supporting the accuracy of our automated segmentation program (fig. S7). Manual segmentation values are available in dataset S2.

### Trait collection

#### Colony size data

To examine the relationship between cuticle investment and colony size, we compiled data from primary literature and online databases, resulting in 2249 records spanning 1617 valid species across 214 genera. For each species, the colony size was calculated as the median of available entries, and genus-level values were calculated as the median across species. In most sources, the reported colony size reflects worker counts from a single nest and does not account for the colony structure that may exist. While this is often a reasonable approximation, it may underestimate the true colony size in species that exhibit polydomy, a colony structure where multiple, spatially separated nests belong to a single colony. Unfortunately, systematic data on polydomy are lacking for most ant species. Polydomy is present all over the ant phylogeny and is often variable even within species. It can depend on geography or even seasonality (*62*, *63*). As such, we cannot determine which species in our dataset are polydomous nor estimate what proportion of our data may be affected. Given this uncertainty, we treat nest size as a proxy for colony size throughout this study. This choice reflects the structure of the available data rather than an ideal metric. While this approach may underestimate colony size in some species, we expect this to reduce the power to detect relationships rather than generate spurious ones. Entry-level details and references are provided in dataset S3.

#### Morphological defensive traits

To explore the potential correlation between a decrease in cuticle thickness and alterations in other morphological defensive traits, we gathered information on the presence of cuticular spines on the mesosoma—a trait recognized as a direct defense against vertebrates or invertebrates (*36*, *37*). Given the presumed evolutionary stability of this trait (*25*), we compiled data at the genus level from Blanchard and Moreau (*25*) and extrapolated it to the species level. Missing data (*n* = 5) were manually retrieved from the image database of Antweb.org using the methodology established by Blanchard and Moreau (*25*). We standardized the number of body parts carrying spines as ordinal data (0: no spines; 1: one pair of spines; 2: two or more pairs of spines). Entry details are provided in dataset S4.

#### Climatic data

To evaluate the impact of climatic factors on cuticle investment, we calculated the mean annual temperature and precipitation from the geographic distribution of each species. We used the GABI data from Kass *et al.* (*64*) whose coordinates were cleaned and snapped to the nearest Worldclim grid cell. Using the R package terra (version 1.7-21) (*65*), we linked each species’ specimen coordinates with Worldclim 2 climatic data (*66*) to obtain mean annual temperature and annual temperature records at a precision of ~1 km^2^. We then calculated the mean values for each species. Entry details are provided in dataset S5.

#### Diet

To assess the effect of the diet on cuticles, we collected data for four main ecological diets of ants: predator (species primarily foraging for prey), omnivore (species foraging for plant-derived food such as honeydew and prey), herbivore [species rarely including prey in their diet), and fungivore (species of the fungus-growing ant clade (*48*)]. Data were collected at the genus level from Blanchard and Moreau (*25*) and extrapolated at the species level. For genera of our dataset with intrageneric diet diversity (*Neoponera*, *Pseudomyrmex*, *Pogonomyrmex*, and *Tetraponera*), we scored species individually from information from the literature. For *Pseudomyrmex* and *Tetraponera*, species were considered herbivorous when exhibiting a deep relationship with a symbiote involving nutritive aspects and omnivorous otherwise. *Pseudomyrmex fiebrigi*, with nothing known about its ecology, was removed from our statistical analysis. Entry details are provided in dataset S6.

#### Foraging strata

We examined the effect of the foraging environment by testing the impact of hypogeic and arboreal foraging strategies in comparison to epigeic foraging. We used the genus-level dataset compiled by Lucky *et al.* (*67*), updated to reflect the latest taxonomic revisions. Genera were classified as hypogeic or arboreal if these were their exclusive foraging strategies and were contrasted with the more ubiquitous epigeic strategy. Detailed entries are provided in dataset S7.

### Phylogenetic data

We used the phylogenetic data produced by Economo *et al.* (*68*) as a reference for phylogeny. Specifically, these data include (i) a maximum clade credibility (MCC) backbone tree reconstructed from the sequence alignment of 679 specimens, (ii) 200 topologies in which almost all (~15,000) ant species were grafted around the leaves of the same genus from the backbone tree, and (iii) one MCC tree with the ~15,000 grafted species. Species names were updated according to the latest taxonomic revisions (AntCat.org, release March 2023).

Given that our database of 3D reconstructed ants holds new species and individuals not identified at the species level (*26*), we had to make some inferences to include these individuals in our phylogenetic analysis. Individuals referenced as “cf.” or “aff.” before their species name were reasonably associated with these species. Unidentified individuals were regrouped under the same name when collected from the same colony. For the ancestral state reconstruction, the species of our dataset missing in the grafted trees were included at the position of another unused species of the same genus.

### Phylogenetic sensitivity analysis

To assess the robustness of our phylogenetic comparative analyses (described below) to alternative phylogenetic frameworks, we conducted a sensitivity analysis using a recently published phylogenetic tree. This tree was inferred with MCMCTree under a likelihood approximation, incorporating a root age uniform prior with soft bounds at 129 to 158 Ma (million years ago) and an independent-rates clock model (*34*). Our datasets were pruned to match the species included in this tree. Because the alternative tree contains only one species per genus, we replaced the species of the tree absent in our dataset by a randomly selected species of the same genus present in our dataset. We then repeated the Bayesian reversible-jump MCMC, PGLS, and phylogenetic path analyses using the same model structure to assess the robustness of the results to the phylogeny used.

### Allometric investigation and caste investment correlations

To explore the scaling rule of cuticles on body volume and differences between castes, we used SMA regression using the R package SMATR—a tool tailored for allometric analysis (*69*). While ordinary least-square regressions entail fitting a line that minimizes errors along the *y* axis, SMA is a symmetric linear regression minimizing errors from both *y* and *x* axes. We examined the allometric growth relationship between log_10_-transformed cuticle volume and body volume and tested for allometric differences between castes and subcastes. Allometric differences between subcastes of workers were analyzed through a subset of 14 species for which we had data for both minor and major workers. These allometric examinations allowed us to calculate the size-standardized cuticle investments as the residuals from SMA regression of log_10_-transformed mean cuticle volume and body volume per species, fitted independently for each caste.

### Modeling trait evolution

To investigate the evolutionary dynamics of size-standardized cuticle investment over the diversification of ants using the MCC backbone tree whose leaves are fully supported by molecular data, we assessed the phylogenetic signal in the data by calculating Pagel’s lambda and Blomberg’s *K* with the R package phytools (version 1.2.0) (*70*). We tested the fit of nine models of trait evolution. We applied a Brownian motion model, a single-optimum Ornstein-Uhlenbeck model, an early burst model, a white noise model, a rate trend model, a lambda model, a kappa model, and a delta model of trait evolution using the function fitContinuous of the R package Geiger (version 2.0.7) (*71*). The models were then compared by their average Akaike information criterion (AIC) and likelihood (table S1).

### Ancestral state estimation and evolutionary shifts

To capture the evolutionary pattern of the size-standardized cuticle investment in ant workers, we estimated the ancestral states of the cuticle investment on the grafted MCC tree. Ancestral state reconstructions were performed using the function anc.ML in the R package phytools (version 1.2.0) (*70*), where we estimated evolutionary parameters and ancestral states using likelihood, given a Brownian-motion model of the evolution of continuous traits.

To identify potential evolutionary shifts in cuticle investment, we applied a Bayesian reversible-jump MCMC approach using the R package bayou (version 2.3.0) (*72*). We tested three models of trait evolution with variation in the relationship between log_10_-transformed cuticle volume and body volume: (i) a global intercept and slope model assuming a single allometric regime across the phylogeny; (ii) a model with separate intercepts but a shared slope, allowing shifts in baseline trait values while maintaining a common allometric relationship; and (iii) a fully flexible model with both separate intercepts and slopes, permitting multiple independent evolutionary regimes. Priors were specified for key parameters, including evolutionary rate, selection strength, and allometric coefficients. MCMC chains were run for 1,000,000 generations with a 30% burn-in, and posterior distributions of shift locations and magnitudes were summarized using credible shift sets, retaining shifts with posterior probabilities >0.7. Model fit was assessed via stepping-stone sampling to estimate marginal likelihoods, allowing the comparison of the three evolutionary scenarios. The second model (baseline shifts) was retained as it showed the highest marginal likelihood, with values as follows: model i, 335.38; model ii, 380.28; model iii, 370.49.

### Correlation between traits

We performed PGLS analysis to examine the relationship between traits, accounting for the nonindependence of data as a result of the shared evolutionary history of the species (i.e., phylogenetic relatedness). PGLS analysis incorporates a covariance matrix to weigh least squares, integrating phylogenetic relatedness into the analysis. This approach assumes that branch length is proportional to the residual error in the model (*73*, *74*). Accordingly, we conducted a PGLS analysis using the gls function from the R package nlme (version 3.1-155) (*75*) on the mean covariance matrix between species, calculated from 200 grafted phylogenetic trees. Because the intragenus relationships were grafted randomly, this approach accounts for species relatedness at the genus level while preserving the species-level detail.

PGLS analysis was performed on a subset of 325 species (133 genera) (fig. S5) that have data for all our tested variables. We tested the effect of these variables on the mean log_10_(cuticle volume) per species while including the mean log_10_(body volume) as a covariate to account for the allometry (*76*). Species’ diet and foraging microhabitat were used as categorical variables with omnivorous and epigeic as references, respectively. Quantitative variables (i.e., log_10_-transformed colony size, climatic data, and spine number) were centered and scaled to make the effects of different variables comparable in magnitude. We ran the first model including all our predictors [i.e., mean annual precipitation, mean annual temperature, log_10_(colony size), spine number, species diet, foraging microhabitat, and log_10_(body volume)]. On the basis of this model, we used the stepAIC function in the R package MASS (version 7.3-55) (*77*) to compare models via stepwise addition and removal of predictors and then run our PGLS analysis a second time with only the predictors identified relevant by the stepAIC function to refine the estimated parameters (see table S2). We compared the role played by variables in the likelihood of our models by running a model pruned of each variable and calculated their log-likelihood contribution as $100-\frac{\text{logLik variable pruned model}}{\text{logLik full model}}\times 100$.

To investigate the association between larger colony size and reduced worker size, we used a similar PGLS approach on a dataset excluding major and media workers, aiming to capture minimal worker body volume per species. We tested log_10_(body volume) against log_10_(colony size) using colony size data at both the species and genus levels across two independent models (see table S3) and including the mean annual temperature to account for any climate-colony size correlations (*7*, *41*, *42*).

To disentangle the causal relationships between cuticle investment, colony size, and species diet, we constructed and compared four plausible evolutionary models using phylogenetic path analysis (*78*) implemented in the R package phylopath (version 1.1.3) (*79*). This method allows the comparison of models of possible causal relationships between traits while testing for direct or indirect effects using the d-separation method (*80*, *81*) and considering the nonindependence of the traits as a result of phylogeny through PGLS analysis. Given the need for maximum resolution to untangle the relationship between cuticle investment and colony size, we used species-level log_10_(colony size) data from a dataset of 172 species (103 genera). Model fits were compared using the CICc calculated as follows

$$\text{CICc}=C+2q\;\frac{n}{n-q-1}$$

where *C* is Fisher’s C-statistic, *n* is the sample size, and *q* is the number of parameters (total number of variables and the number of edges linking them). CICc is conceptually analogous to Corrected Akaike Information Criterion (AICc) but adapted for the C-statistic framework used in phylogenetic path analysis (*78*, *82*, *83*). To account for phylogenetic uncertainties, we estimated model parameters over 200 grafted trees and retrieved models with substantial support (ΔCICc <2 of the best model), following the standard threshold used for information-theoretic approaches and recommendation (*78*, *79*, *84*). From this distribution of fitted models, we analyzed model selection frequency and average model rank on the basis of weights following the approach of Matte and LeBoeuf (*4*). We used the function average_multiphylo edited by Matte and LeBoeuf (*4*) from the phylopath package to handle a set of trees instead of a single one and estimate coefficients of variables’ relationships arising from model selection over the 200 grafted trees. The model weights and *P* values represented in Fig. 3B are the parameters estimated on the MCC grafted tree.

### Diversification rate inference and statistical tests

We sought to infer lineage-specific rates of speciation from our phylogenies and test for correlations with cuticle investment, colony size, and body volume. We used the net diversification rates calculated by Economo *et al.* (*68*) using the Bayesian analysis of macroevolutionary mixtures (*85*) over a set of 400 all-ant phylogenies, using constant-rate macroevolutionary models and focusing on net diversification (not speciation and extinction). Diversification rates were averaged at the genus level, and we tested the dependency of diversification rate on worker cuticle investment, log_10_(colony size), and log_10_(worker body volume). To capture minimal body size, we used log_10_(worker body volume) from our dataset, excluding major and media workers (*11*).

Our analysis used ES-sim, a simulation-based approach that assesses species-specific diversification rates (or “tip rates”) using Pearson’s correlations to detect continuous trait-dependent diversification (*86*, *87*). ES-sim has been shown to be particularly effective for continuous trait-dependent diversification, with greater robustness against false positives compared to formal state-dependent models such as QuaSSE (*86*). ES-sim includes the relatedness of taxa through a matrix of covariance. As such, we used the mean covariance matrix between species calculated from the 200 topologies in our test run with 1,000,000 simulations.

## Supplementary Materials

### This PDF file includes:

Figs. S1 to S7 Tables S1 to S5 Legends for separate files
