# Supplementary material for: The evolution of cheaper workers facilitated larger societies and accelerated diversification in ants
Source: Sci Adv. 2025 Dec 19;11(51):eadx8068. doi: 10.1126/sciadv.adx8068 (PMC12716387; doi:10.1126/sciadv.adx8068)
Supplement: Supplementary file 1 — Figs. S1 to S7 Tables S1 to S5 Legends for separate files [file sciadv.adx8068_sm.pdf]

Science Advances
AAAS

Supplementary Materials for

The evolution of cheaper workers facilitated larger societies and accelerated diversification in ants

Arthur Matte *et al.*

Corresponding author: Arthur Matte, matte.arthur01@gmail.com; Evan P. Economo, economo@umd.edu

Sci. Adv. **11**, eadx8068 (2025)
DOI: 10.1126/sciadv.adx8068

This PDF file includes:

Figs. S1 to S7
Tables S1 to S5
Legends for separate files

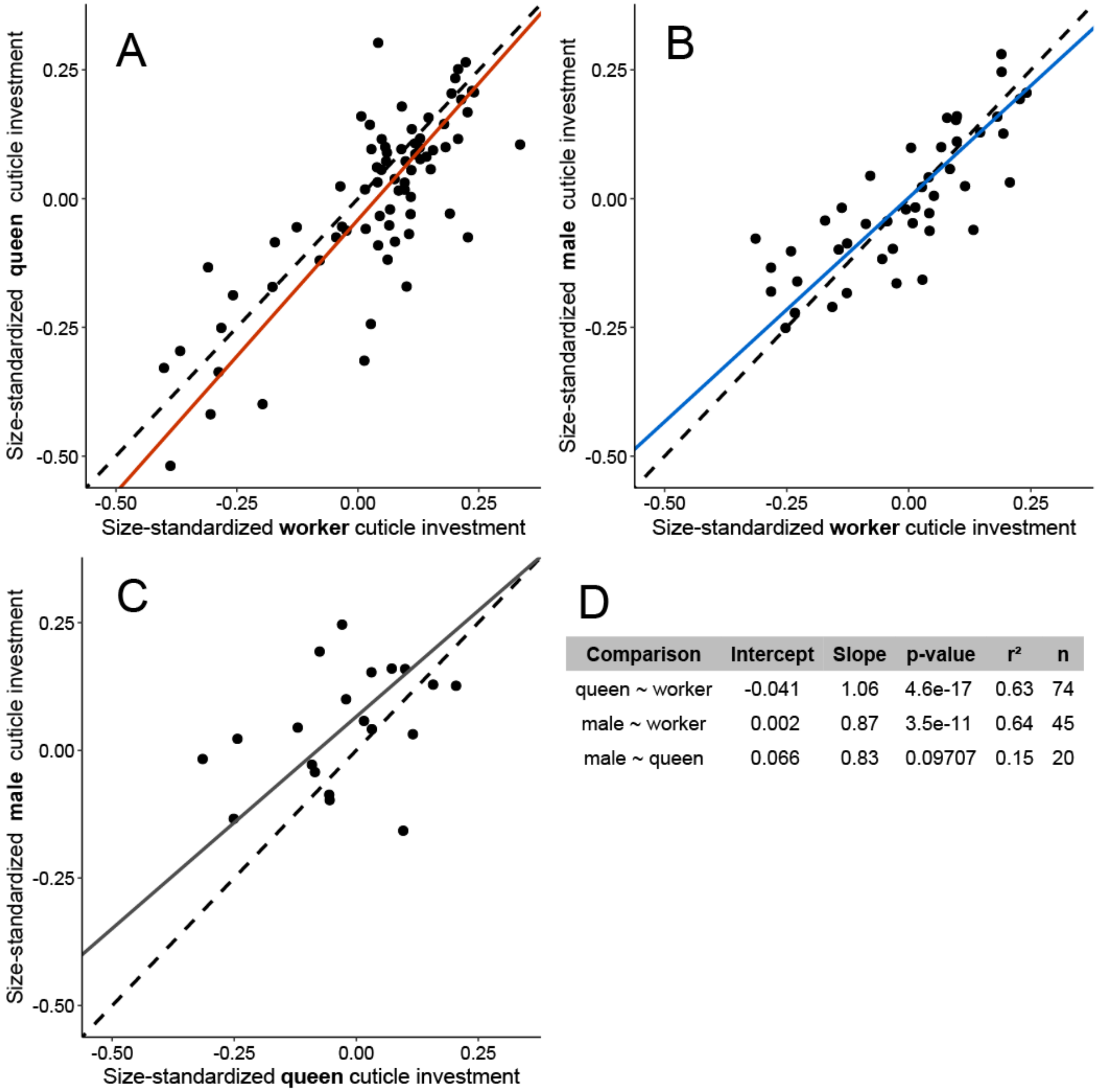

Comparison	Intercept	Slope	p-value	r²	n
queen ~ worker	-0.041	1.06	4.6e-17	0.63	74
male ~ worker	0.002	0.87	3.5e-11	0.64	45
male ~ queen	0.066	0.83	0.09707	0.15	20

Fig. S1.

Intraspecific correlation between caste of size-standardized cuticle investment. Each point in panels **A–C** represents the average size-standardized cuticle investment per species per caste. The table (**D**) summarizes the regression parameters obtained from Standardized-Major-Axis regression and the dashed line represents y = x.

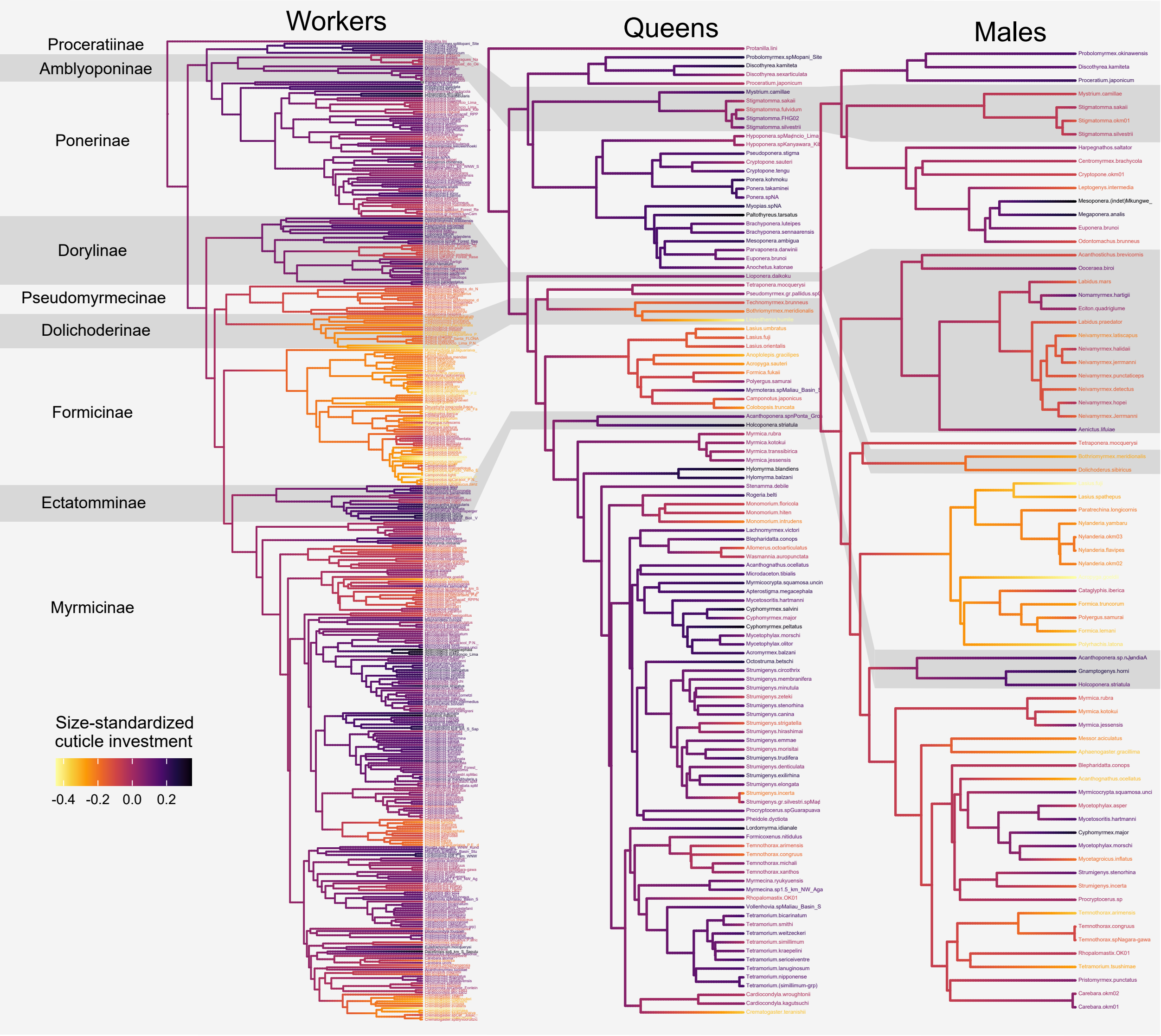

Fig. S2.
Ancestral state reconstruction of the size-standardized cuticle investment for workers, queens, and males with species details. Colored branches indicate predicted values.

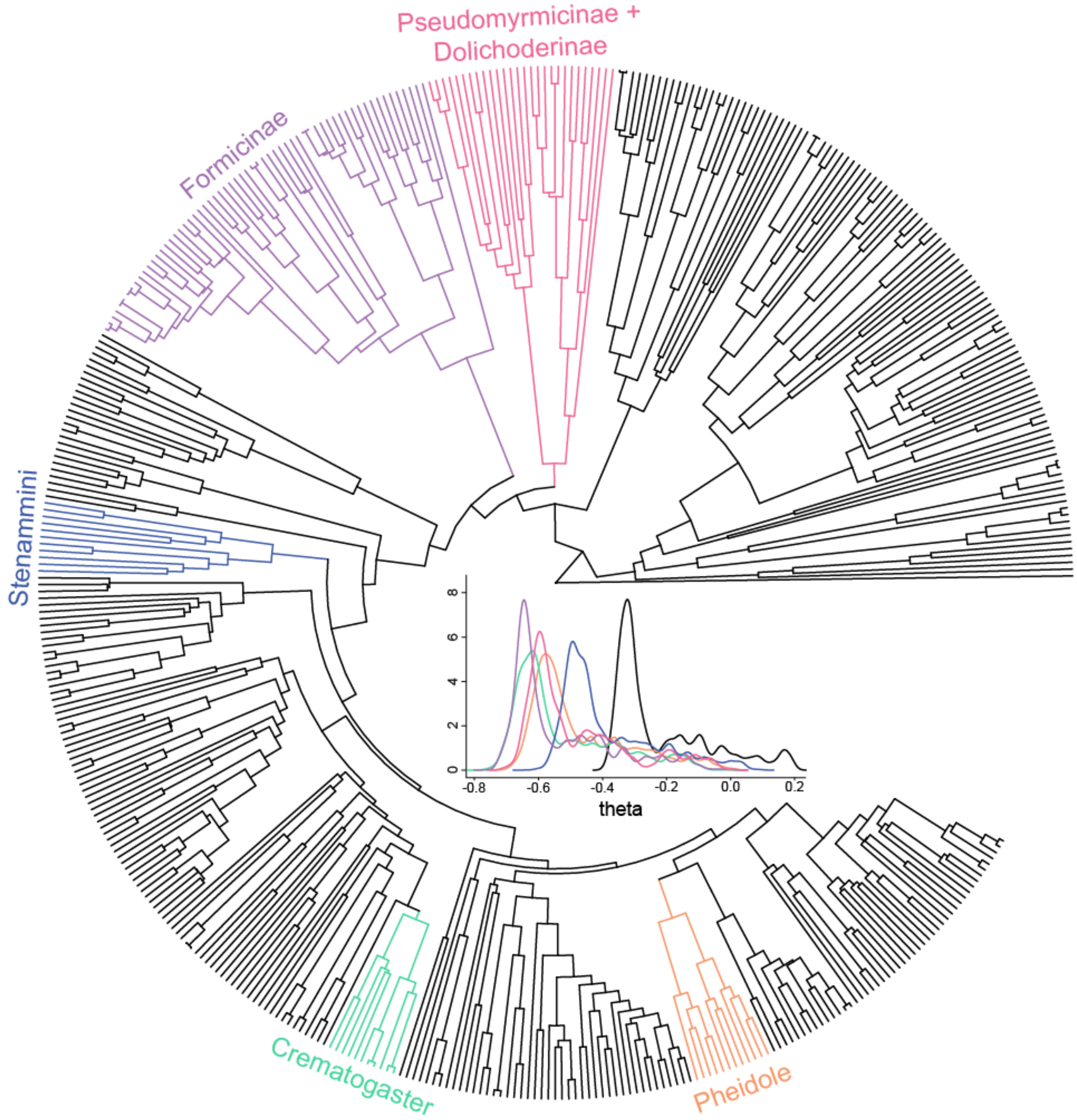

Fig. S3.

Bayesian estimation of evolutionary shifts in worker cuticle investment. Ants phylogeny with branches colored according to inferred grade shifts in size-standardized cuticle investment. Bayesian reversible-jump MCMC analysis identified significant shifts (posterior probability > 0.7) at several key nodes, including the roots of Formicinae (purple), Pseudomyrmecinae + Dolichoderinae (pink), Pheidole (orange), Stenammini (blue), and Crematogaster (green). The inset density plot represents posterior distributions of primary optima (θ) inferred across the phylogeny, with colored peaks corresponding to different evolutionary regimes.

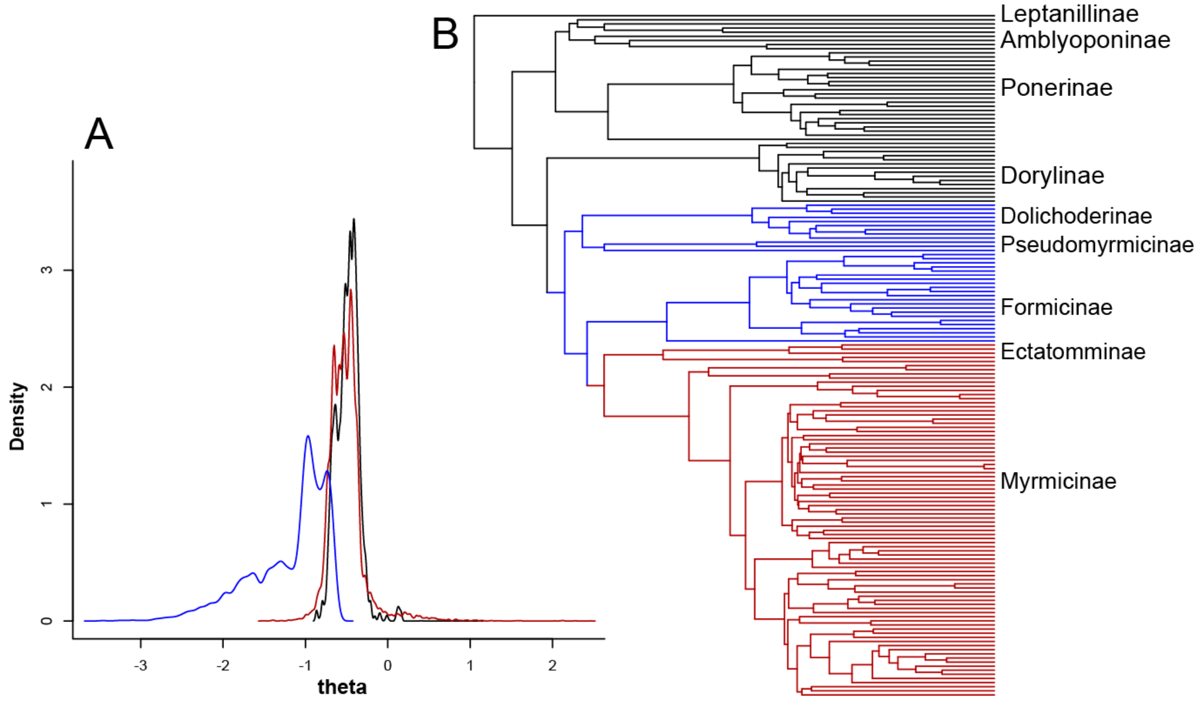

Fig. S4.

The same analysis as presented in Fig. S3 but using an alternative genus-level phylogenetic tree to assess the robustness of our findings across different phylogenetic frameworks. A) Density plot represents the posterior distributions of primary optima (θ) inferred across the phylogeny, with colored peaks corresponding to different evolutionary regimes. B) Ants phylogeny with branches colored according to inferred grade shifts in size-standardized cuticle investment. Major subfamilies are labelled.

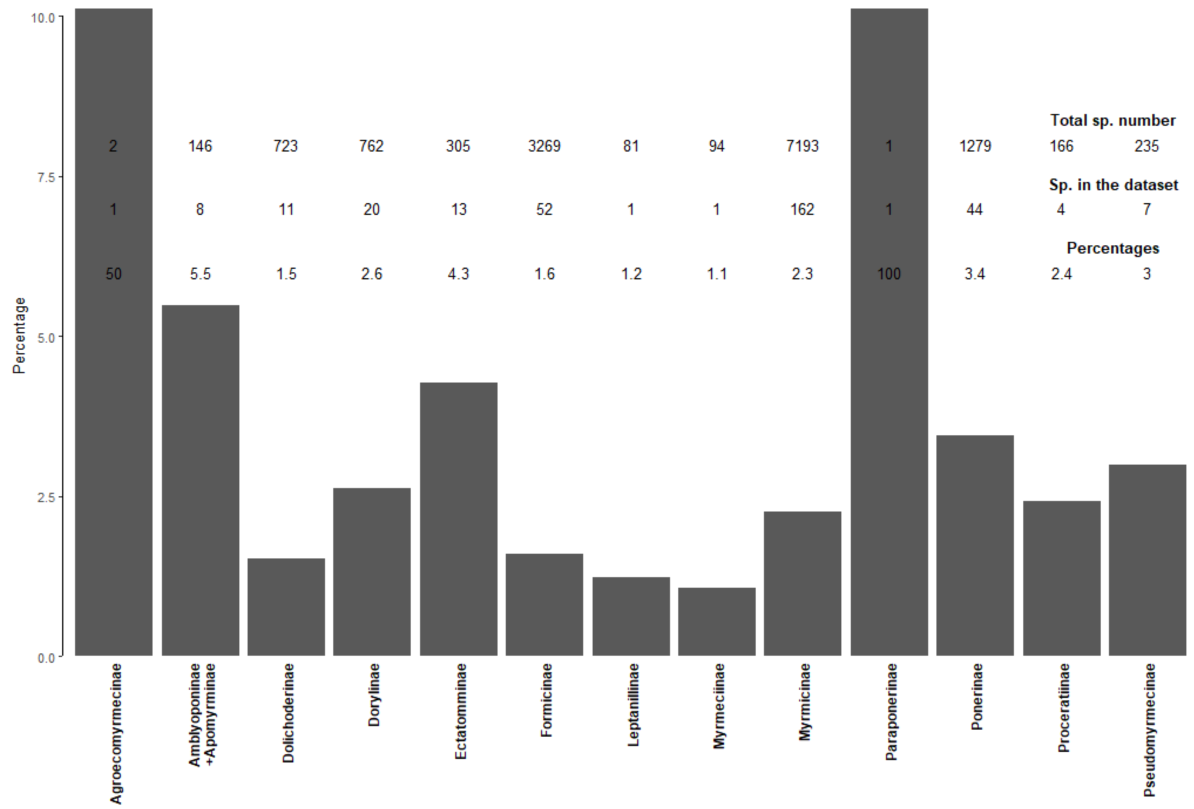

Fig. S5.

Subfamily representation in our dataset of 325 species for Phylogenetic Generalized Least Squares (PGLS) analysis. The total number of species data is sourced from Antcat.org (as of July 2024).

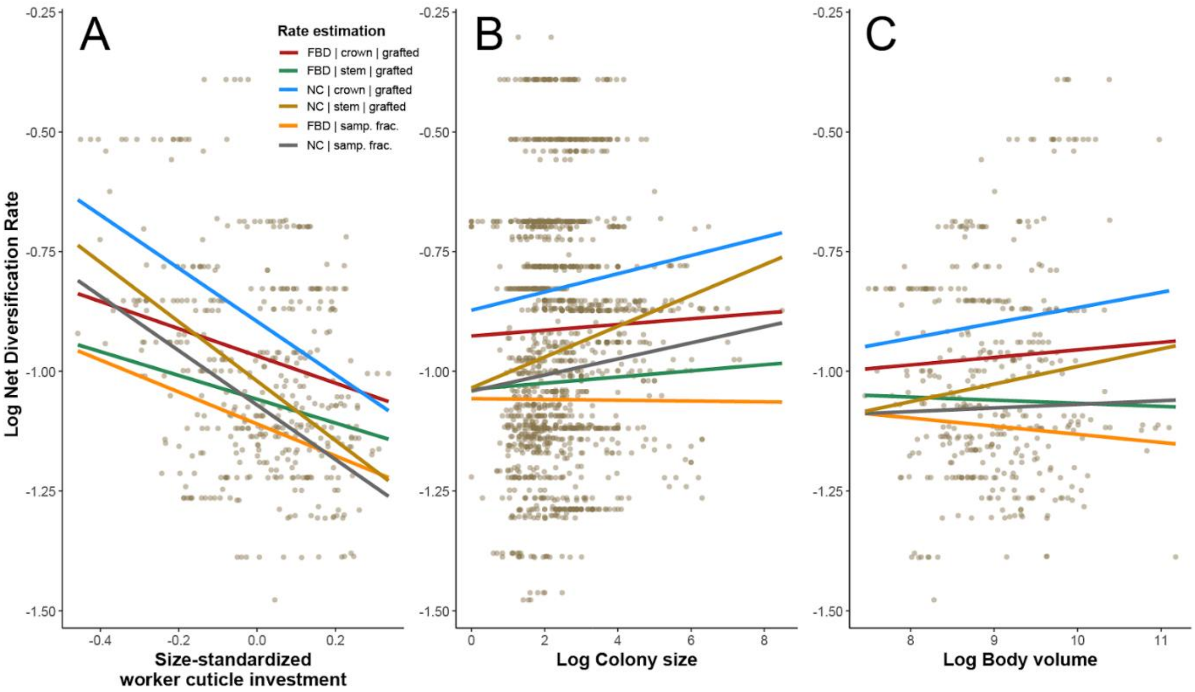

Fig. S6.

Ant log Net diversification rates according to **A**) size-standardized cuticle investment, **B**) colony size, and **C**) body volume with details of variation across rate estimation methods. The displayed dots correspond to the mean diversification rate values obtained from six different methods. FBD (Fossilized Birth-Death) and NC (Node Calibration) refer to the two grafting methods used, while stem and crown denote the two dating methods. Net diversification rates were also inferred using BAMM (Bayesian Analysis of Macroevolutionary Mixtures) directly on the phylogenetic backbone, employing the clade-specific sampling fraction option ("samp. frac."). Lines represent the calculated slope for each method.

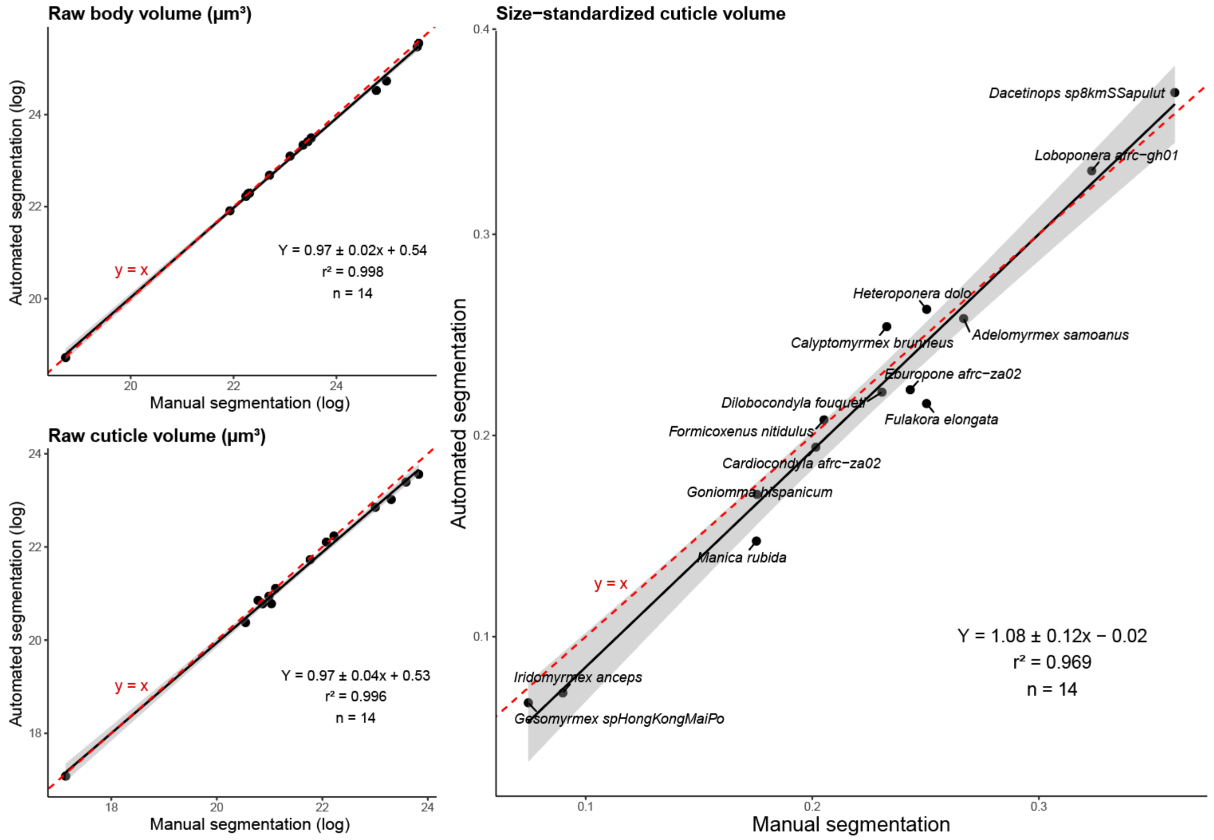

Fig. S7.

Performance evaluation of the automated segmentation program. Comparison of cuticle-to-body volume ratios and raw volume estimates obtained using our unsupervised segmentation program versus manual segmentation across 14 individuals. The dashed line indicates the identity line ($y = x$), and shaded areas represent the 95% confidence intervals.

Table S1.
Evolutionary model of cuticle investment in ants. Comparison of model fits for various models of continuous trait evolution and estimation of phylogenetic signal for size-standardized cuticle investment. Parameters were estimated on a subset of species with phylogenetic relationships supported by molecular data. Data for queens and males are included for reference but should be interpreted cautiously due to the limited species representation.

	Model comparison log-likelihood difference (AIC difference)									Phylogenetic signal			
**Caste**	Brownian motion	Ornstein-Uhlenbeck	early burst	white noise	rate trend	mean trend	lambda	kappa	delta	λ	K	σ^2	n
workers	**0(0)**	0(2)	0(2)	-13(25)	0(2)	0(2)	0(1)	0(2)	0(2)	0.92	0.897	1.6E-04	71
queens	**0(0)**	0(2)	0(2)	-3(6)	0(1)	0(2)	0(2)	0(2)	0(2)	1.06	1.016	3.4E-04	20
males	-1(2)	0(2)	-1(4)	**0(0)**	0(3)	-1(4)	0(2)	-1(4)	0(2)	0	0.81	1.4E-04	15

Table S2.

Detailed results for Phylogenetic Generalized Least Squares (PGLS) correlation analysis of the cuticle investments with other traits. Model 1 incorporated all variables, while Model 2 included only the variable deemed relevant by the stepwise Akaike Information Criterion (stepAIC) for refined parameter estimations. The effect of variables on $\log_{10}$ cuticle volume was tested, with $\log_{10}$ body volume included to address allometric scaling between cuticle volume and body volume. Subfamily representation details in our datasets for each caste are provided in Figure S2.

Included variable		PGLS log cuticle volume ~ variables					
		variable	Value	Std.Error	t.value	p.value	LogLik contribution
Model 1 df = 314		Hypogaeic vs. epigaeic	0.010064	0.022603	0.445257	0.656441	-
		Arboreal vs. epigaeic	0.010952	0.026313	0.416231	0.677525	-
		Mean precipitation	0.001185	0.006247	0.189703	0.849664	-
	Model 2 df = 317	Mean temperature	0.017323	0.006501	2.664733	**0.008099**	1.19084976
		Predator vs. Omnivore	0.074119	0.019992	3.707379	**0.000247**	3.64727956
		Herbivore vs. Omnivore	0.048647	0.035339	1.376567	0.169618	3.64727956
		Fungivore vs. Omnivore	0.140459	0.039077	3.594461	**0.000377**	3.64727956
		Spine number	0.013082	0.006685	1.956818	0.051246	0.645446081
		Log Colony size	-0.040914	0.007961	-5.138942	**4.84E-07**	4.301544657
		Log Body volume	0.97366	0.010901	89.315486	**8.94E-227**	175.4824633

Table S3.

A similar analysis to Table S2, conducted using an alternative phylogenetic tree to assess the robustness of our findings across different phylogenetic frameworks.

Included variable		PGLS log cuticle volume ~ variables					
		variable	Value	Std.Error	t.value	p.value	LogLik contribution
Model 1 df = 117		Hypogaeic vs. epigaeic	0.035697	0.031218	1.143494	0.255168	-
		Arboreal vs. epigaeic	-0.028205	0.038205	-0.738244	0.461844	-
		Mean precipitation	0.009338	0.01318	0.70845	0.480075	-
	Model 2 df = 120	Mean temperature	0.018125	0.011034	1.642548	**0.103095**	1.551000574
		Predator vs. Omnivore	0.148077	0.024642	6.009026	**2.06E-08**	22.94256955
		Herbivore vs. Omnivore	0.040869	0.049602	0.823938	0.411609	22.94256955
		Fungivore vs. Omnivore	0.194471	0.039433	4.93163	**0.000003**	22.94256955
		Spine number	0.014245	0.01123	1.26845	0.207093	0.92910398
		Log Colony size	-0.051183	0.01119	-4.574141	**1.17E-05**	11.21152741
		Log Body volume	0.988587	0.016065	61.534902	**1.29E-92**	242.9614221

Table S4.

Details of the correlation between worker body volume, colony size and mean annual temperature. Results are presented according to two statistical approaches to test correlation: PGLS (Phylogenetic Generalized Least Square), taking account of the phylogeny, and OLS (Ordinary Least Square). Genus and species level correspond to the level at which colony size data have been calculated and applied to the species of our dataset. 'n' refers to the number of species included in the regressions.

Log Body volume ~ Log Colony size + Mean annual temperature							
**Colony size data**	**Methode**	**Variables**	**Value**	**Std.Error**	**t-value**	**p-value**	**n**
Species level	PGLS	Log Colony size	0.1286	0.0382	3.3655	**0.0010**	163
		Mean temperature	0.0065	0.0095	0.6846	0.4946	
	OLS	Log Colony size	0.0891	0.0466	1.9139	0.0574	163
		Mean temperature	0.0020	0.0103	0.1972	0.8439	
Genus level	PGLS	Log Colony size	0.0700	0.0295	2.3693	**0.0184**	321
		Mean temperature	-0.0013	0.0070	-0.1862	0.8524	
	OLS	Log Colony size	0.0591	0.0280	2.1104	**0.0356**	321
		Mean temperature	-0.0070	0.0079	-0.8885	0.3749	

Table S5.
Details of trait-dependent diversification rate analysis. Analyses were conducted with ES-Sim across various dating and tree reconstruction methods with clade grafting or incomplete sampling fractions to represent undiscovered and diversity. The rates were log10 transformed in the tests. Values are estimated slopes and p-values. Net diversification rates were averaged at the genus level. FBD (Fossilized Birth-Death) and NC (Node Calibration) refer to the two grafting methods used, while stem and crown denote the two dating methods. Net diversification rates were also inferred using BAMM directly on the phylogenetic backbone, employing the clade-specific sampling fraction option ("samp. frac.").

	Diversification rate estimation method							
	**FBD | crown grafted**	**NC | crown grafted**	**FBD | stem grafted**	**NC | stem grafted**	**FBD samp. frac.**	**NC samp. frac.**	**Mean all methods**	**n**
Size-standardized cuticle investment	-0.197 | 0.0621	-0.359 | 0.0039	-0.208 | 0.0695	-0.457 | 4e-05	-0.262 | 0.0286	-0.379 | 0.0012	-0.354 | 0.0035	434
Log Colony size	0.004 | 0.9852	0.078 | 0.71418	0.040 | 0.82319	0.143 | 0.50600	0.009 | 0.96422	0.083 | 0.67153	0.065 | 0.7458	1616
Log Body volume	0.103 | 0.3520	0.126 | 0.3809	-0.022 | 0.8571	0.11 | 0.4491	-0.048 | 0.7178	0.026 | 0.8542	0.081 | 0.5687	424

slope | p-value

Legends for separate files (available at the Zenodo repository https://doi.org/10.5281/zenodo.15546461)

Python scripts. Automated segmentation and data extraction scripts for processing 3D μ-CT scans.

R scripts. Statistical and phylogenetic comparative analysis scripts used in this study.

Dataset S1. Extracted cuticle and body volume measurements for all scanned specimens.

Dataset S2. Manually segmented cuticle and body volume measurements for validation of the automated segmentation method.

Dataset S3. Colony size data.

Dataset S4. Presence of cuticular spines on the mesosoma at the genus level.

Dataset S5. Climatic data, including mean annual temperature and precipitation from species distribution records.

Dataset S6. Dietary classification of species and genus into four ecological categories: predator, omnivore, herbivore, and fungivore.

Dataset S7. Foraging strata classification including hypogeic, arboreal and epigeic strategies at the genus level.

Segmentation results. Segmented images generated by our program for automatic segmentation.
